# Livin’ La Vida Sola: Network Diversity and Well‐Being in Middle‐Aged Adults Living Alone

**DOI:** 10.1111/jopy.12998

**Published:** 2024-11-27

**Authors:** Philipp Kersten, Marcus Mund, Franz J. Neyer

**Affiliations:** ^1^ Department of Psychology Friedrich Schiller University Jena Jena Germany; ^2^ Psychological Assessment and Personality Psychology University of Klagenfurt Klagenfurt Austria

**Keywords:** cross‐lagged panel model, dynamic panel model, dynamic transactions, living alone, midlife, network diversity, random‐intercept cross‐lagged panel model, well‐being

## Abstract

**Background:**

For individuals living alone, having a diverse personal network is considered crucial for mitigating the risk of social isolation and enhancing well‐being. Although a reciprocal dynamic between network diversity and well‐being is likely, longitudinal evidence supporting reciprocal effects is limited. This study investigates dynamic transactions between network diversity and well‐being (life satisfaction, loneliness, and depressiveness) in a community‐based sample of middle‐aged adults from Germany. It also explores moderations by the duration of living alone.

**Method:**

Data were drawn from the three‐wave RIKSCHA (Risks and Chances of Living Alone) project, which includes *N* = 389 middle‐aged adults living alone.

**Results:**

Cross‐lagged panel models revealed high rank‐order stabilities and correlated changes in network diversity and well‐being. Random‐intercept cross‐lagged panel models and dynamic panel models indicated that unobserved traits accounted for these high stabilities. Correlated changes disappeared when accounting for the trait‐like stability of variables. Across all models, no evidence of reciprocal associations between network diversity and well‐being was found. All results remained consistent regardless of the duration of living alone.

**Conclusions:**

The study discusses trait factors accounting for the high stabilities observed in network diversity and well‐being among middle‐aged adults living alone. Future research should further explore the traits impacting successful adaptation to living alone within the context of personal networks.

## 1 Introduction

Living alone is often considered a risk factor for social isolation, which can threaten mental health and well‐being (Bennett and Dixon 2006; de Jong Gierveld, Dykstra, and Schenk 2012; Dean et al. 1992; Gaymu and Springer 2012; Holt‐Lunstad et al. 2015). However, not all individuals living alone are equally at risk, as there are significant differences in the structural diversity of their personal networks (Djundeva, Dykstra, and Fokkema 2019; Kersten, Mund, and Neyer 2024). Cross‐sectional research suggests that more diverse networks are associated with better health and well‐being (e.g., Fiori, Antonucci, and Cortina 2006; Litwin and Shiovitz‐Ezra 2010). Thus far, however, there is limited evidence for longitudinal associations between network diversity and well‐being. To address this research gap, the present study investigated longitudinal associations between network diversity and well‐being in a community‐based sample of middle‐aged adults living alone. We hypothesized that network diversity and well‐being would reciprocally influence each other through dynamic‐transactional processes. Additionally, we explored whether individual differences in the duration of living alone moderated these associations, expecting that these dynamic transactions would weaken for individuals who had been living alone for longer periods.

### 1.1 Individual Differences in Network Diversity

Living alone has become increasingly common among middle‐aged adults in recent decades (Demey et al. 2013; Kersten, Mund, and Neyer 2024). As a result, there is growing research interest in understanding how individuals living alone can successfully adapt to this lifestyle. To counter the adverse effects of social isolation that can result from living alone, it is important to have strong personal networks (Bennett and Dixon 2006). Personal networks are characterized by complex, interconnected structural patterns that vary significantly between individuals (Ali et al. 2022). As a result, several researchers have adopted a typological network perspective, seeking to condense multiple structural characteristics into distinct network types (see Antonucci et al. 2010). Numerous studies conducted in Europe, Asia, and the United States have consistently identified two anchor types representing opposite ends of a common dimension. At one end, *more diverse networks* are characterized by their large size, frequent contacts, and heterogeneous composition (i.e., a wide variety of social roles). At the other end, *less diverse networks* are smaller, with infrequent contacts and a more homogeneous composition (Ali et al. 2022; Cheng et al. 2009; Djundeva, Dykstra, and Fokkema 2019; Fiori, Antonucci, and Akiyama 2008; Fiori, Antonucci, and Cortina 2006; Fiori, Smith, and Antonucci 2007; Kersten, Mund, and Neyer 2024; Kim et al. 2017; Litwin 2001; Litwin, Levinsky, and Schwartz 2020; Litwin and Shiovitz‐Ezra 2006, 2010, 2011; Litwin and Stoeckel 2014; Park et al. 2018; Park, Smith, and Dunkle 2014; Sung et al. 2022; Windsor et al. 2016). Recognizing this dimensionality, it is conceivable that every personal network falls somewhere on this scale, reflecting individual differences in *network diversity*.

In previous research, network diversity was often quantified by counting the number of different social roles (e.g., family members, friends, neighbors, or coworkers) within an individual's network (e.g., Ali et al. 2018). However, the present study adopts a broader perspective on network diversity, incorporating structural characteristics commonly examined in typological studies, including *network size*, *number of daily contacts*, and *compositional heterogeneity*. Together, these structural features capture individual differences in the risk of social isolation (Shankar, Rafnsson, and Steptoe 2015).

### 1.2 Network Diversity and Well‐Being

Social relationships are widely recognized as strong determinants of life quality (Holt‐Lunstad, Smith, and Layton 2010). More diverse networks offer greater opportunities for social engagement, participation, and support, all of which contribute to well‐being (Ali et al. 2018; Dembo et al. 2023; Litwin and Shiovitz‐Ezra 2010). Cross‐sectional studies suggest that individuals with more diverse networks consistently report higher life satisfaction, lower levels of loneliness, and less depressive mood compared to those with less diverse networks (e.g., Cheng et al. 2009; Fiori, Antonucci, and Cortina 2006; Litwin and Shiovitz‐Ezra 2010). Longitudinal research further indicates that greater network diversity is associated with more pronounced increases in health and well‐being. Additionally, individual differences in well‐being were found to be associated with differential changes in network diversity (Ali et al. 2023; Dembo et al. 2023; Huxhold, Fiori, and Windsor 2013; Li and Zhang 2015; Lin and Lachman 2023).

Although longitudinal evidence points to reciprocal associations between network diversity and well‐being, most existing research has focused on older adults, leaving a gap in studies on midlife. Additionally, previous research has largely concentrated on general populations, with limited focus on individuals living alone. To address these gaps, the present study investigated reciprocal associations between network diversity and well‐being over time in middle‐aged adults living alone.

### 1.3 Dynamic Transactions Between Network Diversity and Well‐Being in Middle‐Aged Adults Living Alone

According to the paradigm of dynamic transactionism (Magnusson 1990), an individual's personality and social environment are connected through dynamic and reciprocal influences over time, co‐developing through continuous transactions (Mund and Neyer 2014; Neyer and Asendorpf 2001). On one hand, individuals actively select and modify their social environments in ways that align with their personalities. At the same time, they are influenced or socialized by their experiences within these environments (Neyer et al. 2014). These reciprocal influences are referred to as personality–relationship transactions (Allemand and Martin 2016; Neyer et al. 2014). The dynamic‐transactional paradigm posits stability and change of personality and social relationships. Changes occurring over longer periods may result from the influence of personality on social relationships or from the impact of relationships on personality (Mund and Neyer 2014). Several key aspects are important to consider when studying dynamic transactions in the context of the present study.

First, individual lifestyles are often reflected in the social networks to which people belong (Neyer et al. 2014). While those who live alone face a higher risk of social isolation and its negative impact on well‐being compared to people living with others, we propose that this risk is more strongly defined by the structural diversity of an individual's social network than by their living arrangements. Thus, living alone can be seen as a distinct way of regulating social relationships, offering a unique context for studying dynamic transactions between network diversity and well‐being.

Second, while the dynamic transactional paradigm is widely used as a framework for examining personality development (e.g., Neyer and Asendorpf 2001), we argue that its applicability extends to exploring the reciprocal influences between network diversity and well‐being. Research suggests that well‐being exhibits moderate stability across adulthood (Baird, Lucas, and Donnellan 2010), which may allow network diversity to influence well‐being. In contrast, our current understanding of the stability of network diversity is limited. As individuals progress through various life stages embedded in a network of personal relationships (Antonucci et al. 2010), network diversity may display some temporal stability. In fact, a few studies suggest that structural features of personal networks tend to remain stable over longer periods (Antonucci, Ajrouch, and Webster 2019; Dembo et al. 2023; Wrzus et al. 2013).

Third, research indicates that personality–relationship transactions can be particularly pronounced during life transitions (Neyer et al. 2014; Parker et al. 2012; Specht, Egloff, and Schmukle 2011). Therefore, we expected that the dynamic transactions between network diversity and well‐being would differ between individuals who have recently transitioned to living alone and those who have lived alone for longer periods. For instance, someone who has recently started living alone may need time to adjust to this new situation. They may have relocated due to a job change or a divorce, necessitating the establishment of new connections and the reorganization of their personal network in a new environment. This initial phase could feel lonely and frustrating, making it crucial to connect with others. As a result, network diversity and well‐being may be less stable among those with shorter durations of living alone, while reciprocal associations between these domains could be stronger. In contrast, consider someone who has lived alone for decades. This person may have developed resilience against the potential risks of social isolation and may have established a personal network over the years that suits their specific needs. Consequently, the stabilities of network diversity and well‐being might be higher among individuals who have lived alone for longer periods, with weaker reciprocal associations between these domains.

### 1.4 Present Study

The present study investigated reciprocal associations between network diversity and well‐being over time. We expected that (a) network diversity and well‐being would exhibit moderate rank‐order stabilities, (b) individuals with more diverse networks would experience more pronounced increases in life satisfaction and stronger decreases in depressiveness and loneliness compared to individuals with less diverse networks, (c) individuals lower in life satisfaction and higher in loneliness and depressiveness would experience more pronounced decreases in network diversity compared to individuals with higher life satisfaction and lower levels of loneliness and depressiveness, and (d) changes in network diversity would be associated with changes in life satisfaction, loneliness, and depressiveness over time. Moreover, we expected that dynamic transactions between network diversity and well‐being would be moderated by individual differences in the duration of living alone. We assumed that the rank‐order stabilities of network diversity and well‐being would increase over longer durations of living alone and that these increases would go hand in hand with decreasing reciprocal associations and associated changes.

The traditional cross‐lagged panel model (CLPM) is commonly used to examine reciprocal effects in longitudinal data. However, the CLPM has recently been criticized for its underlying assumptions. One key assumption is that, after controlling for autoregressive stabilities and reciprocal lagged effects, no stable trait variance or other sources of stability remain in the assessed variables. Given that psychological constructs often exhibit trait‐like properties (Bailey et al. 2023), several researchers have argued that this assumption is untenable, leading to biased parameter estimates in the CLPM (e.g., Hamaker, Kuiper, and Grasman 2015; Lucas 2023). To address this concern, alternative models have been proposed that control for the trait‐like stability of variables. For example, the random‐intercept CLPM (RI‐CLPM) separates stable between‐person differences from wave‐specific within‐person variance (Hamaker, Kuiper, and Grasman 2015). Alternatively, the dynamic panel model (DPM) incorporates trait factors reflecting the accumulating influence of individual differences on observations over time (Dishop and DeShon 2022; Lucas 2023).

In this study, we initially used the traditional CLPM to analyze our data. However, in response to the criticisms of the CLPM, we also applied both RI‐CLPMs and DPMs to assess the robustness of our findings across these models.

## 2 Method

### 2.1 Ethics Statement

The study protocol was approved by the Institutional Review Board of Friedrich Schiller University Jena (FSV 18/43).

### 2.2 Participants and Procedure

Data were drawn from the *Risks and Chances of Living Alone* (*RIKSCHA*) project, a three‐wave longitudinal study of middle‐aged adults living alone, conducted at Friedrich Schiller University Jena, Germany. Eligibility for participation required (a) being aged between 35 and 60 years and (b) living in a single‐person household. To enhance the likelihood of reaching individuals meeting these criteria, we collaborated with Deutsche Post AG. The sampling process involved several steps. First, we defined the inclusion criteria using an online platform. Deutsche Post then used its in‐house data pool and proprietary algorithm to identify addresses that likely matched these criteria. We subsequently drafted invitation letters, which were sent to households in urban areas of Thuringia, Germany. These letters included personalized links to access the online study, which was conducted using the *formr survey framework* (Arslan, Walther, and Tata 2020). Deutsche Post sent out 20,000 letters, of which 5000 could not be delivered due to incorrect addresses, relocations, or decease. A total of 396 participants enrolled, provided informed consent, and completed the surveys at Wave 1 (T1) in 2020. A subsample of these participants completed online diaries over a 21‐day period at the end of T1. The diary survey was administered only at T1. The subsequent waves, T2 and T3, followed the same procedure, with a 1‐year interval between each wave. In 2021 and 2022, 267 and 275 participants completed the surveys, respectively. Participants were awarded a €50 voucher for completing the diary at T1. Furthermore, they received personalized feedback on their personality traits for finishing the survey at T2. Additionally, they were compensated with a €25 voucher for completing the survey at T3.

Out of the initial 396 participants at T1, seven were excluded from the analyses due to flagged response patterns or falling outside the defined age range, resulting in an analysis sample of 389 individuals. The average age of participants at T1 was 47.22 years (*SD* = 7.41), and 63% of the sample were female. About a quarter of the participants (24%) reported being in a partnership. The sample showed considerable diversity in marital status, educational background, and employment. Specifically, 58% of the participants were unmarried, 27% were divorced, 7% were married but separated, 4% were married, 3% were widowed, and 1% were in a registered partnership. Regarding education, 53% of the participants had completed vocational training, while others held university degrees (27%), advanced technical college degrees (13%), or doctoral degrees (3%). Additionally, 2% had not completed vocational education, and 2% preferred not to disclose their qualifications. In terms of employment status, 87% of the participants were employed, 6% were early retirees, 4% were unemployed, 2% were in (secondary) training, and 1% were on parental leave.

### 2.3 Measures

#### 2.3.1 Network Diversity

We used an ego‐centered social network instrument to assess the structural features of participants' personal networks (see Kersten et al. 2023). At T1, participants (*egos*) were asked to list up to 25 people (*alteri*) in their personal networks based on the following criteria: First, each *alter* needed to hold emotional significance for the *ego;* second, the *ego* needed to be in contact with each *alter* at least once a month. After listing their *alteri*, *egos* categorized their relationship with each *alter* by selecting from 11 categories: *partner*, *parent*, *grandparent*, *sibling*, *child*, *kin*, *friend*, *acquaintance*, *neighbor*, *coworker*, or *other*. *Egos* also rated the average frequency of contact with each *alter*, considering both in‐person and device‐mediated interactions, using a 5‐point scale ranging from 1 (*daily*), 2 (*several times a week*), 3 (*several times a month*), 4 (*several times a year*), to 5 (*once a year or less*). This procedure was repeated at T2 and T3, with *egos* referring to the list of *alteri* prepared in the previous wave. *Egos* were then asked whether each *alter* still belonged to their personal network, allowing for changes in the size and composition of their networks over time. *Egos* were given the flexibility to remove or add *alteri* between waves, with the maximum limit consistently set at 25 *alteri*.

These structural network assessments were used to derive the following variables: (a) *network size* (the total number of *alteri* listed per *ego*); (b) *number of daily contacts* (the sum of *alteri* with whom an *ego* reported daily in‐person or device‐mediated contact); and (c) compositional heterogeneity (the number of different relationship types within an *ego's* personal network). Numerous exploratory studies on network typology have demonstrated that these three indicators are most frequently used to identify structural network types (see Table S1). The *network diversity* index was created by first *z*‐standardizing network size, number of daily contacts, and compositional heterogeneity and then averaging these scores per *ego* (see Table S2 for correlations among the indicators). It is important to note that *network diversity* is a formative variable, meaning it is defined by these indicators, in contrast to reflective variables, where the underlying construct causes the indicators. Before conducting any analyses, we validated *network diversity* by examining its correlation with the classification probabilities from a network typology in a previously published article (see Kersten, Mund, and Neyer 2024). We found a strong positive correlation with the probability of having a *diverse network* (*r* = 0.76; see Table S3), supporting the convergent validity of this measure.

#### 2.3.2 Well‐Being

During each wave, participants completed self‐report measures of life satisfaction, loneliness, and depressiveness. Descriptive statistics, stability coefficients, and internal consistencies of these well‐being measures across all three waves are provided in Table S4.

##### 2.3.2.1 Life Satisfaction

We used the Satisfaction with Life Scale (Diener et al. 1985) to measure life satisfaction. Participants rated the five items (e.g., “The conditions of my life are excellent”) on a 7‐point rating scale ranging from 1 (*does not apply*) to 7 (*fully applies*). The scale was highly consistent across waves (*ω*_T1_ = 0.90, *ω*_T2_ = 0.91, and *ω*_T3_ = 0.92).

##### 2.3.2.2 Loneliness

We employed the eight‐item version of the University of California Los Angeles Loneliness Scale (Hays and DiMatteo 1987) to assess loneliness. Participants rated the items (e.g., “I feel isolated from others”) on a 4‐point rating scale ranging from 1 (*does not apply*) to 4 (*fully applies*). Internal consistency remained high across waves (*ω*_T1_ = 0.90, *ω*_T2_ = 0.89, and *ω*_T3_ = 0.90).

##### 2.3.2.3 Depressiveness

We used the 10 trait items of the State–Trait Depression Scale (Krohne et al. 2002) to measure depressiveness. Participants indicated their agreement with the items (e.g., “I feel miserable”) on a 4‐point frequency scale ranging from 1 (*almost never*) to 4 (*almost always*). The scale was highly consistent across waves (*ω*_T1_ = 0.95, *ω*_T2_ = 0.95, and *ω*_T3_ = 0.95).

#### 2.3.3 Duration of Living Alone

At T1, participants reported the duration of their time living alone (i.e., “How long have you been living alone?”) in years. On average, participants had been living alone for 10.54 years (*SD* = 8.67, *Mdn* = 8.86, *Min* = 0.02, *Max* = 50.88).

### 2.4 Analysis Strategy

Analyses were conducted in *R* 4.3.1 (R Core Team 2023), using the packages *lavaan* 0.6‐15 (Rosseel 2012) and *sirt* 3.12‐66 (Robitzsch 2023). Results with *p* < 0.05 were considered statistically significant. Full information on maximum likelihood was used to handle missing data. Additionally, we employed maximum likelihood estimation with robust standard errors and a scaled test statistic to estimate the model parameters. R scripts are available at https://osf.io/yueg3/.

#### 2.4.1 Measurement Invariance

Latent factors were constructed for life satisfaction, loneliness, and depressiveness. For the loneliness and depressiveness scales, each of which consisted of more than five items, we created three parcels per construct. Measurement invariance was tested by applying increasingly strict models across time. Since the final analyses did not include constraints on intercepts or residual variances, the models focused on metric invariance. Model fit was evaluated using the comparative fit index (CFI), root‐mean‐square error of approximation (RMSEA), and standardized root‐mean‐square residual (SRMR). Acceptable fit was defined as CFI ≥ 0.95, RMSEA ≤ 0.08, and SRMR ≤ 0.08. As shown in the Supplemental Materials (see Table S5), the metric measurement models consistently showed a good fit.

#### 2.4.2 Cross‐Lagged Panel Model (CLPM)

**FIGURE 1:**
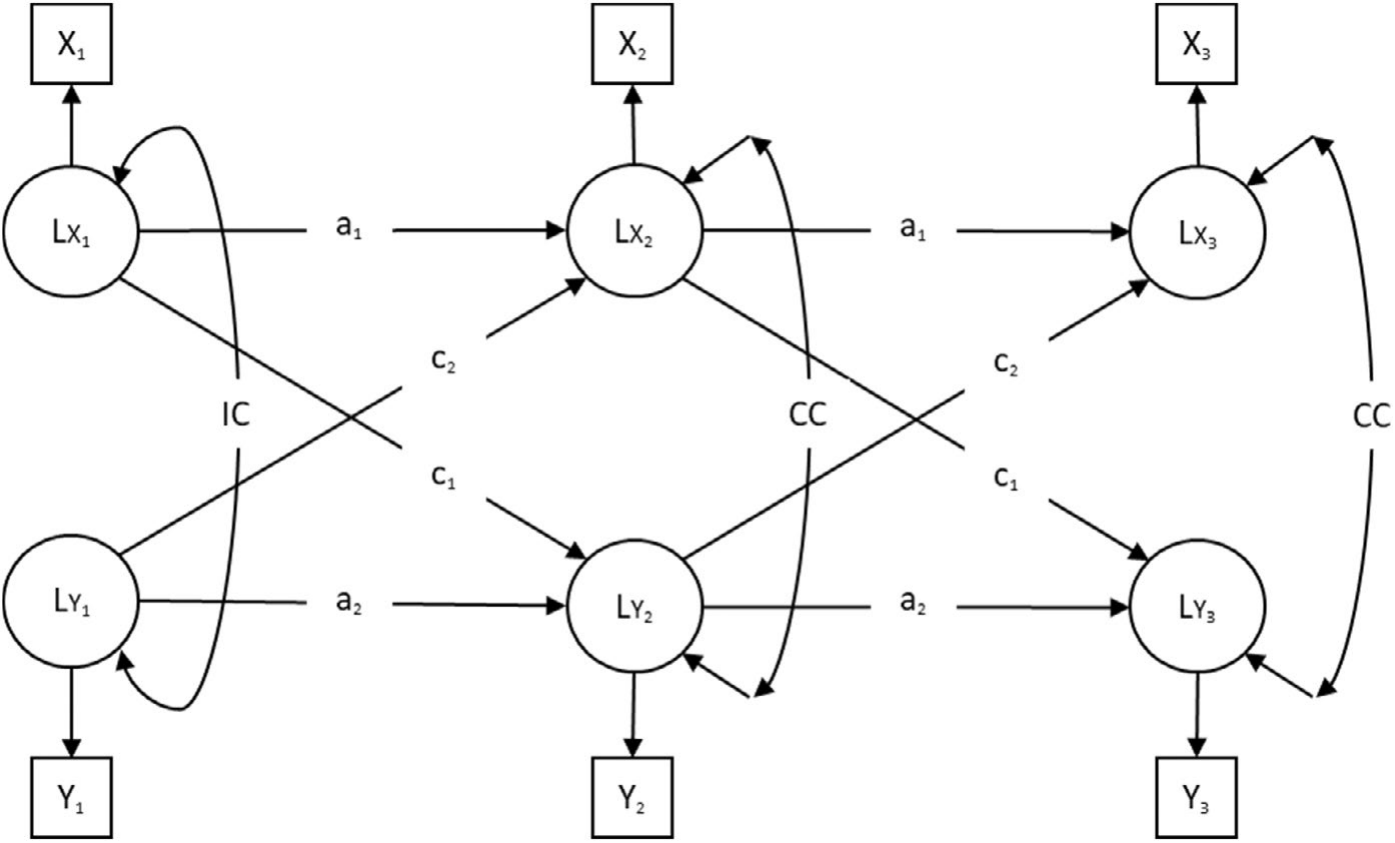
Cross‐lagged panel model (CLPM) for three waves of data. *a* = autoregressive path, *c* = cross‐lagged path, CC = correlated change, Circles = latent variables, Double‐headed arrows = Correlations, IC = initial correlation, Single‐headed arrows labeled with letters = Regressions, Single‐headed arrows without labels = Factor loadings, Squares = manifest indicators. Model parameters with the same label were constrained to equality over time.

We fitted CLPMs within a structural equation framework to investigate the reciprocal associations between network diversity and well‐being over time (see Figure 1). We used measured variables of network diversity and latent variables for the multiple‐indicator constructs of well‐being, fitting separate models for life satisfaction, loneliness, and depressiveness. Assuming stationarity, we constrained the autoregressive paths (*a*_1_ and *a*_2_ in Figure 1) and cross‐lagged paths (*c*_1_ and *c*_2_) to be equal over time. At T1, the variances and covariances of the latent variables (IC) were freely estimated, with equality constraints applied at T2 and T3 (CC). Model comparison tests showed no significant difference in fit between the stationary models and their unconstrained counterparts, supporting the stationarity assumption (see Table S6). Additionally, we applied a lag‐1 structure to the autoregressive and cross‐lagged effects, as well as to the residual covariances. Finally, all models were adjusted for participants' age and gender (1 = female).

#### 2.4.3 Random‐Intercept Cross‐Lagged Panel Model (RI‐CLPM)

**FIGURE 2:**
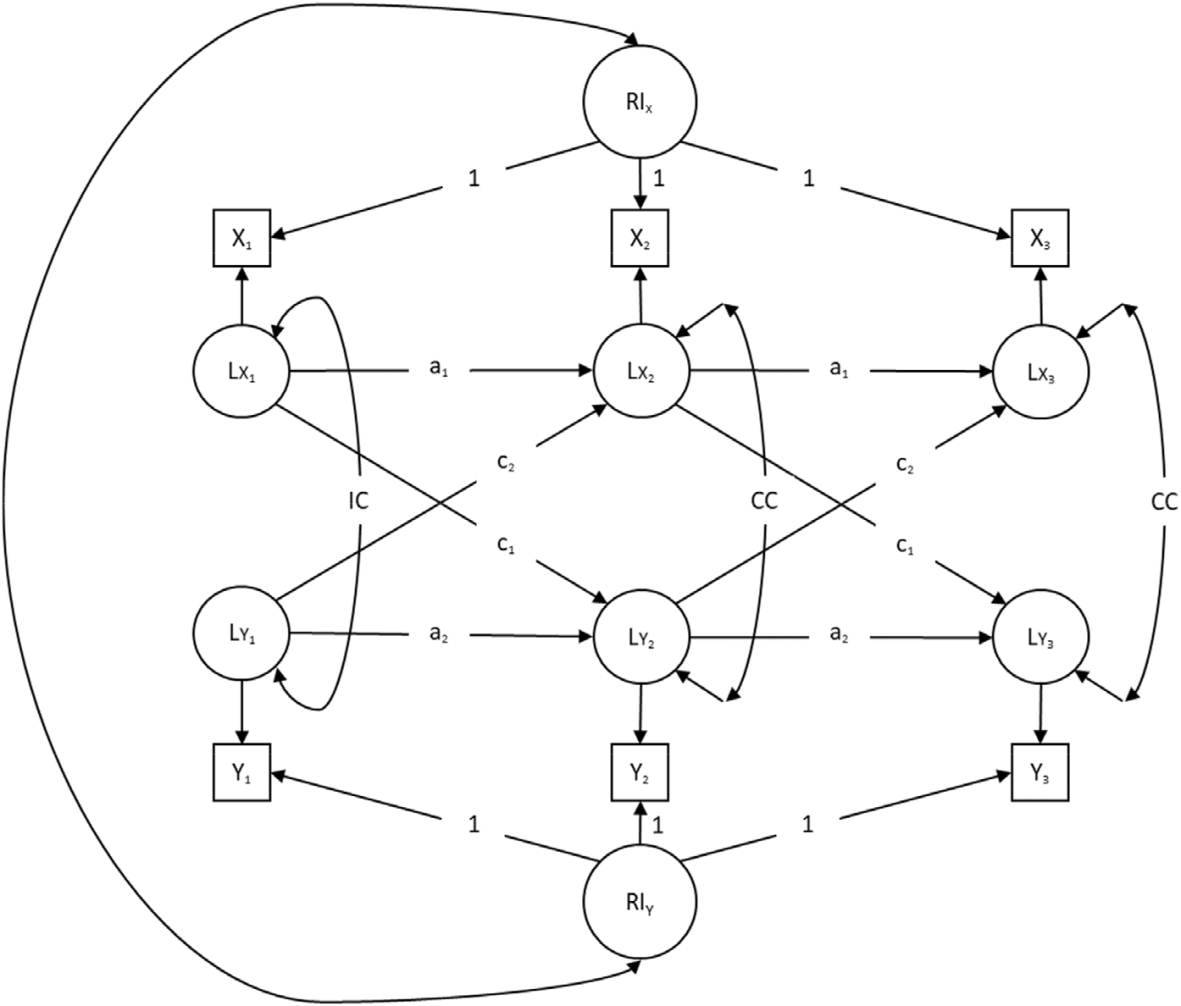
Random‐intercept cross‐lagged panel model (RI‐CLPM) for three waves of data. *a* = autoregressive path, *c* = cross‐lagged path, CC = correlated change, Circles = latent variables, Double‐headed arrows = Correlations, IC = initial correlation, RI = random intercept, Single‐headed arrows labeled with digits or those without labels = Factor loadings, Single‐headed arrows labeled with letters = Regressions, Squares = manifest variables. Model parameters with the same label were constrained to equality over time. Model parameters without labels were freely estimated.

Figure 2 displays a diagram of the RI‐CLPM. In RI‐CLPMs, the autoregressive process was modeled on the residuals of network diversity and well‐being. For this purpose, we used within‐person‐centered variables in the structural part of the models. The models included random‐intercept (RI) factors to reflect the trait‐like stability of the variables over time. Each measurement occasion was specified to load on the RIs of network diversity and well‐being, with factor loadings constrained to 1 across waves. The RIs were not allowed to covary with network diversity and well‐being at T1. However, they were allowed to covary with each other. Additionally, RI‐CLPMs included a similar lag‐1 structure as well as the same covariates and stationarity constraints applied in CLPMs (see Table S7, which indicates no significant difference in fit between the stationary and unconstrained RI‐CLPMs).

#### 2.4.4 Dynamic Panel Model (DPM)

**FIGURE 3:**
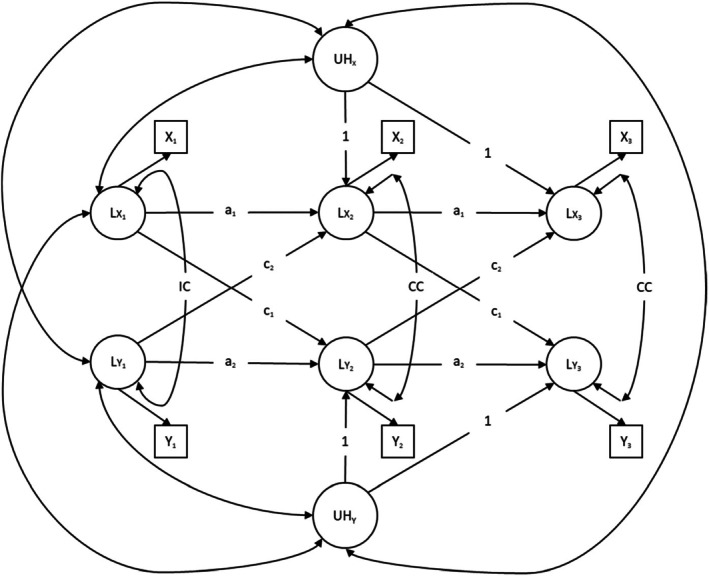
Dynamic panel model (DPM) for three waves of data. *a* = autoregressive path, *c* = cross‐lagged path, CC = correlated change, Circles = latent variables, Double‐headed arrows = Correlations, IC = initial correlation, Single‐headed arrows labeled with digits or those without labels = Factor loadings, Single‐headed arrows labeled with letters = Regressions, Squares = manifest variables, UH = unobserved heterogeneity. Model parameters with the same label were constrained to equality over time. Model parameters without labels were freely estimated.

Figure 3 shows a diagram of the DPM. DPMs included additional latent factors representing the accumulating influence of unobserved heterogeneity (UH) on the observations of network diversity and well‐being over time. The factor loadings were constrained to 1 at T2 and T3 for each UH factor. Moreover, the UH factors were allowed to freely covary with network diversity and well‐being at T1 and with each other. Additionally, DPMs were estimated using the same lag‐1 structure, covariates, and stationarity constraints as applied in CLPMs and RI‐CLPMs (see Table S8, which shows no significant difference in fit between the stationary and unconstrained DPMs).

#### 2.4.5 Model Fit

In addition to using CFI, RMSEA, and SRMR, we used the Akaike Information Criterion (AIC) and Bayesian Information Criterion (BIC) to assess the model fit across CLPMs, RI‐CLPMs, and DPMs. Better model fit is indicated by higher CFI values and lower values for RMSEA, SRMR, AIC, and BIC.

#### 2.4.6 Local Structural Equation Modeling (LSEM)

To explore whether the model parameters of CLPMs, RI‐CLPMs, and DPMs changed over the duration of living alone, we conducted LSEM permutation tests (Olaru et al. 2023). The analyses included values spanning from 0.5 to 20 years of living alone, incrementing in steps of 0.5. Values below 0.5 and over 20 years were excluded due to limited effective sample sizes.

## 3 Results

### 3.1 Preliminary Results

#### 3.1.1 Attrition Analyses

Due to a dropout rate of 30% between waves, we examined differences between continuers and dropouts in the study variables. We found no significant differences in manifest well‐being scores or the duration of living alone (*ds* ≤ 0.18). However, those who continued were older (*M*_*C*_ = 48.23, *M*_*D*_ = 45.51, *d* = 0.37, *p* < 0.001) and had more diverse networks compared with those who dropped out (*d* = 0.28, *p* = 0.006). Additionally, male participants were more likely to drop out than female participants (*χ* ^2^ = 5.14, OR = 1.67, *p* = 0.023). Given that female participants reported having more diverse networks than male participants (see Table 1), the dropout effect associated with network diversity was likely confounded with the gender effect. Nevertheless, the variance of network diversity within the analysis sample (*var* = 0.65) did not drastically differ from that of the panel sample (*var* = 0.70). Thus, we expected no substantial bias in the results.

**TABLE 1:** Descriptive statistics, T1 correlations, and stabilities of the study variables (*N* = 389).

Variable	*M*	*SD*	1	2	3	4	5	6
1. Network diversity	0.00	0.81	***0.89***					
2. Life satisfaction	4.85	1.27	0.06	***0.81***				
3. Loneliness	1.88	0.56	**−0.16**	**−0.59**	***0.77***			
4. Depressiveness	1.84	0.63	−0.09	**−0.70**	**0.66**	***0.83***		
5. Duration of living alone (years)	10.54	8.67	−0.02	**0.14**	0.01	−0.06		
6. Age (years)	47.22	7.41	0.08	**0.11**	−0.07	**−0.10**	**0.27**	
7. Gender (female)	0.63	—	**0.20**	0.02	**−0.13**	−0.02	−0.01	0.01

*Note:* Bold correlations are significant (*p* < 0.05). Italic correlations along the diagonal reflect stabilities between T1 and T2.

#### 3.1.2 Descriptive Results

Table 1 displays the descriptive statistics and correlations of the study variables at T1. Correlations were mostly weak (|0.10| ≤ *r* ≤ |0.27|), whereas the well‐being measures were all strongly correlated (|0.59| ≤ *r* ≤ |0.70|). The stability coefficients between T1 and T2 were consistently high across all study variables (see Table S4 for stabilities between the other waves).

### 3.2 CLPM

Table 2 shows the standardized CLPM results. As indicated by RMSEA, CFI, and SRMR, all three CLPMs demonstrated acceptable to good fit (see Table 5).

**TABLE 2:** Standardized CLPM parameter estimates (*N* = 389).

Network diversity and	(a) Life satisfaction			(b) Loneliness			(c) Depressiveness		
	Estimate	95% CI	*p*	Estimate	95% CI	*p*	Estimate	95% CI	*p*
Autoregressive effects									
*a*_1_ ND_*t*_ → ND_*t*+1_	0.888	[0.864; 0.921]	< 0.001	0.889	[0.865; 0.921]	< 0.001	0.888	[0.865; 0.921]	< 0.001
*a*_2_ WB_*t*_ → WB_*t*+1_	0.865	[0.817; 0.902]	< 0.001	0.865	[0.810; 0.908]	< 0.001	0.871	[0.842; 0.907]	< 0.001
Cross‐lagged effects									
*c*_1_ ND_*t*_ → WB_*t*+1_	−0.024	[−0.070; 0.020]	0.268	0.018	[−0.028; 0.064]	0.441	0.040	[−0.001; 0.081]	0.054
*c*_2_ WB_*t*_ → ND_*t*+1_	0.010	[−0.024; 0.044]	0.554	0.001	[−0.042; 0.043]	0.977	−0.005	[−0.041; 0.032]	0.809
Correlations									
IC ND_*t*1_ ↔ WB_*t*1_	0.056	[−0.037; 0.149]	0.235	−0.134	[−0.228; −0.039]	0.006	−0.078	[−0.181; 0.025]	0.137
CC ND_*t*_ ↔ WB_*t*_	0.143	[0.046; 0.239]	0.004	−0.166	[−0.277; −0.055]	0.003	−0.104	[−0.205; −0.002]	0.045

Abbreviations: CC = correlated change; CFI = comparative fit index, CLPM = cross‐lagged panel model, IC = initial correlation, ND = network diversity, RMSEA = root‐mean‐square error of approximation, SRMR = standardized root‐mean‐square residual, WB = well‐being.

#### 3.2.1 Autoregressive Effects

The autoregressive effects of network diversity (*a*_1_), life satisfaction, loneliness, and depressiveness (*a*_2_) were consistently large and significant, indicating high rank‐order stabilities of network diversity and well‐being.

#### 3.2.2 Cross‐Lagged Effects

No cross‐lagged effects (*c*_1_ and *c*_2_) reached significance, indicating no reciprocal associations between network diversity and well‐being over time.

#### 3.2.3 Initial Correlations and Correlated Changes

At T1, network diversity was negatively correlated with loneliness but was neither correlated with life satisfaction nor with depressiveness. Over time, network diversity was weakly correlated with life satisfaction, loneliness, and depressiveness, indicating that changes in network diversity were associated with changes in well‐being.

### 3.3 RI‐CLPM

Table 3 shows the standardized RI‐CLPM results. As indicated by RMSEA, CFI, and SRMR, all three models demonstrated acceptable to good fit (see Table 5).

**TABLE 3:** Standardized RI‐CLPM parameter estimates (*N* = 389).

Network diversity and	(a) Life satisfaction			(b) Loneliness			(c) Depressiveness		
	Estimate	95% CI	*p*	Estimate	95% CI	*p*	Estimate	95% CI	*p*
Autoregressive effects									
*a*_1_ ND_*t*_ → ND_*t*+1_	0.515	[−0.198; 1.293]	0.211	0.504	[0.177; 0.904]	0.010	0.552	[−0.406; 1.568]	0.308
*a*_2_ WB_*t*_ → WB_*t*+1_	0.498	[0.055; 0.898]	0.024	0.118	[−0.312; 0.558]	0.581	0.360	[−0.027; 0.771]	0.085
Cross‐lagged effects									
*c*_1_ ND_*t*_ → WB_*t*+1_	−0.140	[−0.684; 0.384]	0.599	0.038	[−0.365; 0.446]	0.844	0.061	[−0.55; 0.680]	0.837
*c*_2_ WB_*t*_ → ND_*t*+1_	−0.073	[−0.549; 0.410]	0.778	0.122	[−0.074; 0.333]	0.208	0.016	[−0.408; 0.441]	0.938
Correlations									
IC ND_*t*1_ ↔ WB_*t*1_	−0.087	[−0.825; 0.650]	0.816	−0.239	[−0.542; 0.064]	0.122	−0.157	[−0.555; 0.242]	0.441
CC ND_*t*_ ↔ WB_*t*_	0.084	[−0.304; 0.471]	0.672	−0.002	[−0.249; 0.246]	0.991	−0.062	[−0.513; 0.390]	0.789

Abbreviations: CC = correlated change, CFI = comparative fit index, IC = initial correlation, ND = network diversity, RI‐CLPM = random‐intercept cross‐lagged panel model, RMSEA = root‐mean‐square error of approximation, SRMR = standardized root‐mean‐square residual, WB = well‐being.

#### 3.3.1 Autoregressive Effects

The autoregressive effect of network diversity (*a*_1_) was significant in the loneliness model, whereas it was not significant in the other two models. The autoregressive effect of life satisfaction (*a*_2_) was significant, while those of loneliness and depressiveness were not significant.

#### 3.3.2 Cross‐Lagged Effects

The analyses revealed no significant cross‐lagged effects between network diversity and well‐being (*c*_1_ and *c*_2_).

#### 3.3.3 Initial Correlations and Correlated Changes

Network diversity and well‐being were not correlated at T1, nor were they correlated over time.

#### 3.3.4 Random Intercepts

Table S9 displays the unstandardized RI‐CLPM results. As can be seen, the variance estimates of the RI factors for network diversity and well‐being were consistently significant.

### 3.4 DPM

Table 4 shows the standardized DPM results. As indicated by RMSEA, CFI, and SRMR, all three DPMs demonstrated acceptable to good fit (see Table 5).

**TABLE 4:** Standardized DPM parameter estimates (*N* = 389).

Network diversity and	(a) Life satisfaction			(b) Loneliness			(c) Depressiveness		
	Estimate	95% CI	*p*	Estimate	95% CI	*p*	Estimate	95% CI	*p*
Autoregressive effects									
*a*_1_ ND_*t*_ → ND_*t*+1_	0.410	[0.046; 0.789]	0.027	0.475	[0.111; 0.854]	0.011	0.410	[0.054; 0.782]	0.024
*a*_2_ WB_*t*_ → WB_*t*+1_	0.346	[−0.180; 0.866]	0.200	0.138	[−0.277; 0.555]	0.513	0.328	[−0.174; 0.836]	0.201
Cross‐lagged effects									
*c*_1_ ND_*t*_ → WB_*t*+1_	0.010	[−0.244; 0.265]	0.937	0.021	[−0.279; 0.322]	0.888	−0.085	[−0.342; 0.167]	0.502
*c*_2_ WB_*t*_ → ND_*t*+1_	0.007	[−0.239; 0.252]	0.958	0.149	[−0.036; 0.336]	0.117	−0.029	[−0.237; 0.178]	0.781
Correlations									
IC ND_*t*1_ ↔ WB_*t*1_	0.058	[−0.035; 0.150]	0.224	−0.135	[−0.23; −0.040]	0.005	−0.080	[−0.183; 0.024]	0.130
CC ND_*t*_ ↔ WB_*t*_	0.219	[−0.022; 0.460]	0.075	0.052	[−0.176; 0.280]	0.654	−0.168	[−0.412; 0.077]	0.178

Abbreviations: CC = correlated change, CFI = comparative fit index, DPM = dynamic panel model, IC = initial correlation, ND = network diversity, RMSEA = root‐mean‐square error of approximation, SRMR = standardized root‐mean‐square residual, WB = well‐being.

**TABLE 5:** Model fit indices.

Network diversity and	Model	CFI	RMSEA	SRMR	AIC	BIC
(a) Life satisfaction	CLPM	0.959	0.056	0.042	15,430.345	15,703.832
	RI‐CLPM	0.961	0.055	0.041	15,424.905	15,710.283
	DPM	0.961	0.056	0.041	15,430.203	15,731.435
(b) Loneliness	CLPM	0.953	0.075	0.043	4979.397	5181.539
	RI‐CLPM	0.958	0.072	0.041	4968.517	5182.551
	DPM	0.958	0.075	0.040	4973.191	5203.079
(c) Depressiveness	CLPM	0.978	0.062	0.028	3975.463	4177.606
	RI‐CLPM	0.980	0.059	0.027	3968.066	4182.099
	DPM	0.981	0.061	0.025	3971.089	4200.977

Abbreviations: AIC = Akaike information criterion, BIC = Bayesian information criterion, CFI = comparative fit index, CLPM = cross‐lagged panel model, DPM = dynamic panel model, RI‐CLPM = random‐intercept cross‐lagged panel model, RMSEA = root‐mean‐square error of approximation, SRMR = standardized root‐mean‐square residual.

#### 3.4.1 Autoregressive Effects

The autoregressive effect of network diversity (*a*_1_) was significant in all models. However, the autoregressive effects of life satisfaction, loneliness, and depressiveness (*a*_2_) were not significant.

#### 3.4.2 Cross‐Lagged Effects

We found no cross‐lagged effects between network diversity and well‐being (*c*_1_ and *c*_2_).

#### 3.4.3 Initial Correlations and Correlated Changes

At T1, network diversity was weakly correlated with loneliness but was neither correlated with life satisfaction nor with depressiveness. Over time, we found no correlated changes of network diversity and well‐being.

#### 3.4.4 Unobserved Heterogeneity

Table S10 presents the unstandardized DPM results. As shown, the variance estimates of the UH trait factors for network diversity and well‐being were consistently nonsignificant.

### 3.5 Model Fit

Table 5 presents an overview of fit indices across the models. As shown, CFI values were the highest for RI‐CLPMs and DPMs. RMSEA was the lowest for RI‐CLPMs, while SRMR was the lowest for DPMs. Additionally, AIC values were the lowest for RI‐CLPMs, and BIC values were the lowest for CLPMs. Since RI‐CLPMs showed the best values for the majority of fit indices (i.e., CFI, RMSEA, and AIC), we concluded that RI‐CLPMs demonstrated the best overall model fit.

### 3.6 LSEM

#### 3.6.1 Moderation by the Duration of Living Alone

Results of the LSEM permutation tests are presented in Tables S11 (CLPM), S12 (RI‐CLPM), and S13 (DPM). In the RI‐CLPMs (see Table S12) and DPMs (see Table S13), the cross‐lagged effect of depressiveness on network diversity and their correlated change increased over the duration of living alone. However, the confidence intervals for these effects were large, indicating that they may not be substantial (see Figure S1). Thus, there was no consistent evidence of significant changes in autoregressive or cross‐lagged effects over the duration of living alone.

### 3.7 Summary

Table 6 summarizes the results across the models. The autoregressive effects of network diversity were significant in both CLPMs and DPMs but mostly nonsignificant in RI‐CLPMs. The autoregressive effects of well‐being were consistently significant in CLPMs. In RI‐CLPMs, only the autoregressive effect of life satisfaction was significant, while none of the autoregressive effects for well‐being were significant in DPMs. Across all models, no cross‐lagged effects were found between network diversity and well‐being. Correlated changes in network diversity and well‐being were significant in CLPMs, but they did not reach significance in RI‐CLPMs or DPMs. The variances of the RI factors were consistently significant in RI‐CLPMs. The variances of the UH trait factors were not significant in DPMs. Additionally, the majority of fit indices suggested that RI‐CLPMs had the best model fit. Finally, all results remained consistent regardless of how long individuals had been living alone.

**TABLE 6:** Summary of findings.

Model parameter	CLPM	RI‐CLPM	DPM
Autoregressive effects			
*a*_1_ ND_*t*_ → ND_*t*+1_	✓	×	✓
*a*_2_ WB_*t*_ → WB_*t*+1_	✓	×	×
Cross‐lagged effects			
*c*_1_ ND_*t*_ → WB_*t*+1_	×	×	×
*c*_2_ WB_*t*_ → ND_*t*+1_	×	×	×
Correlations			
CC ND_*t*_ ↔ WB_*t*_	✓	×	×
Trait variance			
ND	NA	✓	×
WB	NA	✓	×

Abbreviations: × = not significant, ✓ = *p* < 0.05, CC = correlated change, CLPM = cross‐lagged panel model, DPM = dynamic panel model, NA = not applicable, ND = network diversity, RI‐CLPM = random‐intercept cross‐lagged panel model, WB = well‐being.

These results indicate that network diversity and well‐being are stable over time, although unmeasured individual differences likely accounted for a significant portion of this stability. There was no evidence of reciprocal influences between these domains. However, we found correlated changes in network diversity and well‐being that disappeared after accounting for unmeasured individual differences. Finally, there was no evidence that the duration of living alone moderated these relationships.

## 4 Discussion

This study examined dynamic transactions between network diversity and well‐being over time among middle‐aged adults living alone. While we found high rank‐order stabilities in both network diversity and well‐being, there was also evidence that unmeasured individual differences accounted for these stabilities. Most importantly, we found no reciprocal associations between network diversity and well‐being. Additionally, although changes in network diversity were associated with changes in well‐being in models that did not control for unmeasured confounders, these correlated changes disappeared after accounting for the trait‐like stability of the variables. Finally, all results remained consistent regardless of how long individuals had been living alone.

### 4.1 Stability and Change of Network Diversity and Well‐Being

This study introduced the structural diversity of personal networks as a trait measure of social embeddedness. More diverse and less diverse networks differ in size, contact frequency, and compositional heterogeneity. This aligns with extensive research on network typology (see Antonucci et al. 2010 for an overview) and supports the idea that network diversity is a valid measure of individual differences in social integration. While less diverse networks may suggest a higher risk of social isolation compared to more diverse networks, it is also possible that some less diverse networks have specific compositions that provide sufficient social integration. For example, some less diverse networks might predominantly consist of close family members who offer adequate support and participation. As a result, individuals in these networks may report relatively high well‐being due to their close engagement and integration. However, recent research on individuals living alone indicates that the prevalence of specific social roles is not correlated with network diversity (see Kersten, Mund, and Neyer 2024). Therefore, network diversity is more likely associated with the variety of social roles rather than the presence of particular roles.

Descriptive analyses revealed that network diversity and well‐being exhibited substantial temporal stability. CLPMs confirmed this, showing significant autoregressive stability in both domains over time. These results suggest that individual differences in network diversity and well‐being are highly stable, aligning with previous research documenting considerable rank‐order stability in these domains during midlife and beyond (Antonucci, Ajrouch, and Webster 2019; Baird, Lucas, and Donnellan 2010; Dembo et al. 2023; Wrzus et al. 2013). However, the autoregressive stability coefficients were lower in RI‐CLPMs and DPMs, suggesting that unmeasured variables with trait‐like features likely account for these rank‐order stabilities. RI‐CLPMs and DPMs represent similar approaches to control for the trait‐like stability of variables. However, they differ in how they model the influence of traits on the autoregressive and cross‐lagged effects, which affects the conceptual meaning of these traits. In RI‐CLPMs, traits have direct temporal effects on observations at each wave. In contrast, DPMs assume that the trait influence accumulates over time, affecting observations both directly and indirectly through all previous waves (Andersen 2022; Lucas 2023; Lüdtke and Robitzsch 2022; Murayama and Gfrörer 2024; Usami 2021; Usami, Murayama, and Hamaker 2019). Consequently, Usami (2021) argued that models incorporating accumulating factors implicitly control for *time‐varying* confounders, while RI‐CLPMs capture *stable traits*. In the present study, the random‐intercept variances of RI‐CLPMs were consistently significant, whereas the variances attributed to unobserved heterogeneity of DPMs were not. Notably, research suggests that RI‐CLPMs may sometimes mistakenly detect stable traits in the presence of time‐varying confounders (Bailey et al. 2023). However, if that were the case in this study, the variances of unobserved heterogeneity would likely have been significant. Therefore, we conclude that stable traits, rather than time‐varying processes, accounted for the high stabilities of network diversity and well‐being. These stable traits could include demographic factors (e.g., gender, age, education, and socioeconomic status) and personal characteristics (e.g., personality, cognitive abilities, motivation, health conditions, and response bias), among others (Andersen 2022; Murayama and Gfrörer 2024). Additionally, middle‐aged adults navigate multiple social roles they are involved in and feel responsible for, including work, family, friendships, and caregiving, uniquely bridging social connections across younger and older generations (Infurna, Gerstorf, and Lachman 2020). Individual differences in how they balance their own needs within these diverse social roles may reflect additional sources of stability. Overall, this study provides detailed insights on the stability of network diversity and well‐being in midlife. While there is evidence of substantial stability in these domains, the present study also highlights the crucial role of unobserved individual differences in shaping this stability. Identifying and understanding these sources of stability presents a promising avenue for future research.

The most central finding of this study is the absence of reciprocal associations between network diversity and well‐being over time. The high stabilities of both variables left very little room for such reciprocal effects to emerge. Individuals enrolled in the study at varying points in their duration of living alone, each reporting a different time frame. This arbitrary selection of assessment points may have contributed significantly to the high stabilities and the absence of reciprocal associations between network diversity and well‐being. Life transitions, such as divorce or the sudden death of a partner, might initially lead individuals to live alone. These transitions could challenge both personal networks and well‐being, potentially setting the stage for dynamic transactions to unfold. To explore the potential impact of such transitions, we examined whether the duration of living alone moderated any of the associations. We anticipated weaker rank‐order stabilities and stronger reciprocal effects between network diversity and well‐being for those who had recently transitioned into living alone. However, all findings remained consistent regardless of how long individuals had been living alone, suggesting that both network diversity and well‐being are largely resilient to recent changes in living arrangements.

An important consideration is the diverse reasons and pathways that prompt individuals to live alone. Some choose this lifestyle voluntarily, while others are compelled by circumstances, such as separation or the death of a partner (Demey et al. 2013). These different pathways can shape how people perceive living alone over time. For instance, those who choose to live alone voluntarily may increasingly value privacy and solitude, which could positively impact their well‐being. Conversely, those who have broken up with a cohabiting partner may long for cohabitation again over time, struggling with extended periods of singlehood. This could negatively impact their well‐being and lead to a gradual withdrawal from personal relationships. Despite these diverse pathways into living alone, there are various other life transitions middle‐aged adults are normatively faced with. These include changes in work (e.g., job loss, promotion, changing companies, and career advancement), physical and mental health (e.g., onset of chronic conditions, declining cognitive abilities, and mental health problems), family dynamics (e.g., parenthood, grandparenthood, and caregiving), and social life (e.g., separation and repartnering) with a concurrent impact on personal relationships and well‐being (Demey et al. 2013; Infurna 2021; Infurna, Gerstorf, and Lachman 2020; Lachman, Teshale, and Agrigoroaei 2015). Additionally, in a recent study, later‐born cohorts of people living alone were found to report lower social well‐being compared to earlier‐born cohorts (Fritsch, Riederer, and Seewann 2023). Altogether, the diversity of pathways into living alone, differential coping strategies, various life transitions, and cohort effects may provide more nuanced insights and contribute to considerable heterogeneity in personal networks and well‐being. Future research should explore these aspects affecting the stability and change of personal networks and well‐being among individuals living alone.

Lastly, CLPMs revealed a consistent pattern of correlated changes, suggesting a co‐development of network diversity and well‐being driven by unknown common causes (Allemand and Martin 2016). This co‐development is thought to contribute to the cumulative stability of these variables (Neyer and Lehnart 2007). Interestingly, after accounting for additional sources of stability, changes in network diversity were no longer associated with changes in well‐being. This suggests that, in CLPMs, network diversity and well‐being may have been confounded by shared variance in stable traits accounting for the cumulative stability and correlated changes. RI‐CLPMs and DPMs captured unmeasured between‐person differences and perhaps the shared variance of network diversity and well‐being, which might partially explain the disappearance of correlated changes. This shared variance could reflect factors that set the course of co‐development. The variety of factors contributing to the stability and change of network diversity and well‐being underscores the heterogeneity of individual circumstances and social experiences shaping the psychological adaptation to living alone (Bennett and Dixon 2006; Demey et al. 2013; Liu et al. 2020). Altogether, this study cannot explicitly rule out the possibility that network diversity and well‐being are reciprocally associated. We contend that the findings from CLPMs, RI‐CLPMs, and DPMs complement each other in demonstrating the complexity of longitudinal associations between the aspects of social integration and psychological well‐being in middle‐aged adults living alone. Future research might further explore factors contributing to the success of living alone within the context of personal networks.

### 4.2 Limitations

Several limitations warrant consideration when interpreting the present findings. First, there was a 30% attrition rate between waves, with a higher likelihood of dropout among male participants and those with less diverse networks. While the variance of network diversity remained unaffected by this attrition, it is crucial to consider the potential impact of attrition on the generalizability of findings.

Second, despite employing an elaborate sampling process, participants self‐selected into the study which possibly introduced sampling bias. This self‐selection might have resulted in a sample that limits the generalizability of our findings to the broader population of middle‐aged adults living alone. Individuals who chose to participate might possess characteristics or experiences that differ systematically from those who opted against participation. However, in an attempt to counterbalance selection bias, we successfully recruited a community‐based sample with heterogeneous sociodemographic backgrounds, aiming to include a reliable representation of middle‐aged adults who live alone.

Third, the three waves of the study covered 2 years. Developmental processes, however, may unfold over more extended periods. Given the potential variability in the subjective experiences and social connections of middle‐aged adults living alone, including shorter and more frequent measurement intervals could provide a more nuanced understanding of dynamic transactions between network diversity and well‐being. Future research could consider adopting a more fine‐grained approach to enhance the temporal resolution in the complex interplay between social networks and well‐being.

Fourth, the data were collected at random points in time, with each individual reporting their unique timeline of living alone. However, research consistently shows that personality–relationship transactions are most pronounced during life transitions (e.g., Mund and Neyer 2014). Consequently, it remains unclear whether the transition to living alone triggers trait changes in social relationships and well‐being. While longitudinal studies with frequent assessments of life transitions are generally rare, some recent studies have addressed this gap by examining the relationship between life events and personality trait changes (e.g., Denissen et al. 2019). Therefore, future studies should aim to track individuals as they transition into living alone to better understand these dynamics.

Fifth, an important aspect of this study is the geographical and demographic focus of the sample, which centers on urban areas in Germany, with populations ranging from roughly 65,000 to 210,000. Consequently, the findings may not generalize to larger metropolitan areas, rural regions, or countries other than Germany. In fact, studies suggest that the correlation between marriage and happiness varies across countries (Lucas and Dyrenforth 2006), likely reflecting cultural differences in societal norms that may shape attitudes toward living alone. In addition, alternative family structures and living arrangements, such as “living‐apart‐together” relationships and the increasing acceptance of the LGBTQIA+ community, have become more prevalent in recent years (Flores 2021; Hagemeyer et al. 2015). Moreover, social life has transformed substantially over the past two decades with technological advancements, including smartphones and social media platforms, which have profoundly impacted personal lifestyles and interpersonal relationships (Fritsch, Riederer, and Seewann 2023). Therefore, it is essential to consider the historical, regional, and cultural contexts when interpreting the findings of the present study.

Finally, the study collected data amid the global spread of the coronavirus. Initially, the pandemic was accompanied by the implementation of strict governmental measures such as physical distancing and reduced social contact to minimize infection numbers. Consequently, individuals, especially those living alone, experienced substantial disruptions in their social lives, with implications for well‐being in the short term (Pauly et al. 2022; Reitsema et al. 2022). Despite potential pandemic influences, the data were collected during the summer months in 2020, 2021, and 2022, respectively. During these periods, social restrictions were largely relaxed. Nevertheless, additional research might offer valuable insights into personal networks and well‐being in post‐pandemic times.

### 4.3 Conclusions

This study examined the psychological well‐being of individuals living alone within the context of their personal networks. The high stability of both network diversity and well‐being highlights the significant role of individual differences in shaping social integration and psychological adaptation to living alone. However, the lack of reciprocal associations between these factors emphasizes the need for a deeper understanding of how personal networks and well‐being co‐develop. Future research should focus on identifying specific traits and life circumstances that influence the psychological adaptation to living alone, offering insights into how well‐being can be fostered in this population.

## Author Contributions

P.K., M.M., and F.J.N. contributed to the study concept and design. Data collection was performed by P.K. P.K. completed statistical analyses and interpretation of results in collaboration with M.M., and F.J.N. P.K. prepared initial manuscript drafts, which were revised and edited by M.M. and F.J.N.

## Conflicts of Interest

The authors declare no conflicts of interest.

## Supporting information


**Data S1.**.

## Data Availability

The data used in the present study are available at https://osf.io/yueg3/.
