# Supplementary material for: Livin’ La Vida Sola: Network Diversity and Well‐Being in Middle‐Aged Adults Living Alone
Source: J Pers. 2024 Nov 27;93(5):1149–63. doi: 10.1111/jopy.12998 (PMC12421717; doi:10.1111/jopy.12998)
Supplement: Supplementary file 1 — Data S1.. [file JOPY-93-1149-s001.docx]

# Livin’ La Vida Sola:

# Network Diversity and Well-Being in Middle-Aged Adults Living Alone

Philipp Kersten^1^, Marcus Mund^2^, and Franz J. Neyer^1^

^1^Friedrich Schiller University Jena, Germany

^2^University of Klagenfurt, Austria

Supplemental Materials

| Table S1. Overview of network-typology studies and most frequently employed structural network-type indicators. | | | |
| --- | --- | --- | --- |
| Study | Network-type indicator | | |
|  | **Network size** | **Compositional heterogeneity** | **Contact frequency** |
| Ali et al. (2022) | Number of core confidants  Number of:  a) family  b) friends | Number of different network relationships | Frequency of contact |
| Cheng et al. (2009) | Network size | – | Frequency of contact with:  a) vertical family  b) horizontal family  c) non-family |
| Djundeva et al. (2019) | Network size | Ties in network:  a) child  b) grandchild  c) sibling  d) parent  e) friend  f) formal helper  g) other | Daily contact |
| Fiori et al. (2006) | Number of children | – | Frequency of contact with:  a) children  b) friends |
| Fiori et al. (2007) | Network size | – | Frequency of contact with:  a) family  b) friends |
| Fiori et al. (2008) | Network size | – | Frequency of contact with:  a) family  b) friends |

| Table S1 *continued*. | | | |
| --- | --- | --- | --- |
| Study | **Network size** | **Compositional heterogeneity** | **Contact frequency** |
| Kersten et al. (2024) | Network size | Ties in network:  a) partner  b) close kin  c) extended kin  d) friend  e) cooperative non-kin | Number of daily contacts |
| Kim et al. (2017) | Number of close ties | Number of:  a) children  b) other immediate family  c) ex-partner  d) friends  e) neighbor | Frequency of contact with:  a) children  b) other immediate family  c) ex-partner  d) friends  e) neighbor |
| Li & Zhang (2015) | Number of close children | – | Play cards, attend organized social activities |
| Litwin (2001) | Proximate children | – | Contact with:  a) children  b) friends  c) neighbors |
| Litwin et al. (2020) | Network size | Ties in network:  a) spouse  b) children  c) other family  d) friends  e) others | Daily contact |
| Litwin & Shiovitz-Ezra (2006) | Number of proximate children | – | Contact with:  a) children  b) friends  c) neighbors |

| Table S1 *continued*. | | | | |
| --- | --- | --- | --- | --- |
| Study | **Network size** | | **Compositional heterogeneity** | **Contact frequency** |
| Litwin & Shiovitz-Ezra (2010) | Number of:  a) children  b) close relatives  c) friends | | – | Frequency of getting together with neighbors, organized group meetings |
| Litwin & Shiovitz-Ezra (2011) | Number of:  a) children  b) close relatives  c) friends  d) neighbors | | – | Frequency of getting together with neighbors |
| Litwin & Stoeckel (2014) | Network size | | Ties in network:  a) spouse  b) children  c) other family  d) friends  e) others | Daily contact |
| Park et al. (2018) | Number of:  a) close family  b) close friends | | – | Frequency of contact with:  a) close family  b) close friends |
| Park et al. (2014) | Family size |  | – | Frequency of contact with:  a) children  b) friends |
| Sung et al. (2022) | Number of:  a) relatives  b) friends | | – | contact frequency:  a) friends  b) relatives |
| Windsor et al. (2016) | Family network size  Friend network size | | – | Family contact frequency  Friend contact frequency |
| *Note.* Dashes denote that there was no measure of this structural characteristic (column) included in the particular study (row). | | | | |

| Table S2. T1 correlations of network diversity indicators (*N* = 389). | | | | |
| --- | --- | --- | --- | --- |
| Variable | *M* | *SD* | 1 | 2 |
| 1. Network size | 8.28 | 6.29 |  |  |
| 2. Number of daily contacts | 1.32 | 1.53 | **.35** |  |
| 3. Compositional heterogeneity | 4.01 | 2.00 | **.78** | **.28** |
| *Note. M* = Mean. *SD* = Standard deviation. Bold correlations are significant at *p* < 0.05. | | | | |

| Table S3. Correlations of network diversity with network-type membership probabilities (*N* = 389). | | | | |
| --- | --- | --- | --- | --- |
| Variable | 1 | 2 | 3 | 4 |
| 1. Network diversity |  |  |  |  |
| 2. Probability: *Diverse* | **.76** |  |  |  |
| 3. Probability: *Partner-focused* | .05 | **-.22** |  |  |
| 4. Probability: *Loose-knit* | -.03 | **-.33** | **-.23** |  |
| 5. Probability: *Restricted* | **-.70** | **-.49** | **-.23** | **-.45** |
| *Note.* Network diversity reflects the mean across the standardized values of network size, number of daily contacts, and compositional heterogeneity. Variables 2 - 5 characterize the classification probabilities of the network typology developed by Kersten et al. (2024). Bold correlations are significant (*p* < .01). | | | | |

| Table S4. Descriptive statistics of the study variables over time. | | | | | | | | | | | | | | | |
| --- | --- | --- | --- | --- | --- | --- | --- | --- | --- | --- | --- | --- | --- | --- | --- |
| Variable | ω | | |  | *M* | | |  | *SD* | | |  | Stability | | |
|  | T1 | T2 | T3 |  | T1 | T2 | T3 |  | T1 | T2 | T3 |  | T1 → T2 | T2 → T3 | T1 → T3 |
| Network diversity | 0.79 | 0.79 | 0.79 |  | 0.00 | 0.00 | 0.00 |  | 0.79 | 0.79 | 0.78 |  | 0.89 | 0.91 | 0.84 |
| Life satisfaction | 0.90 | 0.91 | 0.92 |  | 4.85 | 4.84 | 4.86 |  | 1.27 | 1.30 | 1.32 |  | 0.81 | 0.81 | 0.72 |
| Loneliness | 0.90 | 0.89 | 0.90 |  | 1.88 | 1.91 | 1.89 |  | 0.56 | 0.55 | 0.55 |  | 0.77 | 0.80 | 0.72 |
| Depressiveness | 0.95 | 0.95 | 0.95 |  | 1.84 | 1.81 | 1.83 |  | 0.63 | 0.63 | 0.63 |  | 0.83 | 0.83 | 0.77 |
| *Note.* ω = McDonald’s omega. *M* = Mean. *SD* = Standard deviation. Network diversity reflects the mean across three standardized variables, including *network size*, *number of daily contacts*, and *compositional heterogeneity*. Life satisfaction was measured on a 7-point Likert-type scale. Loneliness and depressiveness were measured on 4-point Likert-type scales, respectively. | | | | | | | | | | | | | | | |

| Table S5. Measurement invariance tests over time. | | | | | | |
| --- | --- | --- | --- | --- | --- | --- |
| Measure | χ^2^ (df) | Δχ^2^ *p* | RMSEA [90% CI] | SRMR | CFI | ΔCFI |
| *Life Satisfaction* | | | | | | |
| Configural invariance | 141.216 (72) |  | 0.050 [0.037; 0.062] | 0.025 | 0.981 |  |
| **Metric invariance** | **147.631 (80)** | **0.672** | **0.047 [0.035; 0.058]** | **0.031** | **0.982** | **0.001** |
| Scalar invariance | 154.781 (90) | 0.711 | 0.043 [0.031; 0.054] | 0.032 | 0.982 | 0.000 |
| Residual invariance | 168.245 (100) | 0.528 | 0.042 [0.031; 0.053] | 0.034 | 0.981 | 0.001 |
| *Loneliness* | | | | | | |
| Configural invariance | 22.111 (15) |  | 0.035 [0.000; 0.064] | 0.024 | 0.996 |  |
| **Metric invariance** | **23.909 (19)** | **0.771** | **0.026 [0.000; 0.054]** | **0.026** | **0.998** | **0.002** |
| Scalar invariance | 28.767 (25) | 0.555 | 0.020 [0.000; 0.047] | 0.028 | 0.998 | 0.000 |
| Residual invariance | 31.444 (31) | 0.941 | 0.006 [0.000; 0.039] | 0.028 | 1.000 | 0.002 |
| *Depressiveness* | | | | | | |
| Configural invariance | 16.481 (15) |  | 0.016 [0.000; 0.052] | 0.012 | 1.000 |  |
| **Metric invariance** | **18.775 (19)** | **0.701** | **0.000 [0.000; 0.044]** | **0.019** | **1.000** | **0.000** |
| Scalar invariance | 26.654 (25) | 0.242 | 0.013 [0.000; 0.043] | 0.023 | 0.999 | 0.001 |
| Residual invariance | 30.463 (31) | 0.814 | 0.000 [0.000; 0.037] | 0.024 | 1.000 | 0.001 |
| *Note.* df = Degrees of freedom. RMSEA = Root mean square error of approximation. SRMR = Standardized root mean square residual. CFI = Comparative fit index. Bold print indicates the selected level of invariance. | | | | | | |

| Table S6. CLPM stationarity tests. | | | | | | |
| --- | --- | --- | --- | --- | --- | --- |
| *Network diversity* and | χ^2^ (df) | Δχ^2^ *p* | RMSEA [90% CI] | SRMR | CFI | ΔCFI |
| a) *Life satisfaction* | | | | | | |
| Unconstrained model | 336.236 (149) |  | 0.057 [0.049; 0.065] | 0.041 | 0.959 |  |
| **Stationary model** | **343.932 (156)** | **0.644** | **0.056 [0.048; 0.064]** | **0.042** | **0.959** | **0.000** |
| b) *Loneliness* | | | | | | |
| Unconstrained model | 189.890 (56) |  | 0.078 [0.066; 0.091] | 0.041 | 0.954 |  |
| **Stationary model** | **199.072 (63)** | **0.457** | **0.075 [0.063; 0.086]** | **0.043** | **0.953** | **0.001** |
| c) *Depressiveness* | | | | | | |
| Unconstrained model | 151.519 (56) |  | 0.066 [0.054; 0.079] | 0.027 | 0.977 |  |
| **Stationary model** | **155.706 (63)** | **0.886** | **0.062 [0.049; 0.074]** | **0.028** | **0.978** | **0.001** |
| *Note.* CLPM = Cross-lagged panel model. df = Degrees of freedom. RMSEA = Root mean square error of approximation. SRMR = Standardized root mean square residual. CFI = Comparative fit index. Bold print indicates the selected model. | | | | | | |

| Table S7. RI-CLPM stationarity tests. | | | | | | |
| --- | --- | --- | --- | --- | --- | --- |
| *Network diversity* and | χ^2^ (df) | Δχ^2^ *p* | RMSEA [90% CI] | SRMR | CFI | ΔCFI |
| a) *Life satisfaction* | | | | | | |
| Unconstrained model | 326.206 (146) |  | 0.056 [0.048; 0.065] | 0.040 | 0.961 |  |
| **Stationary model** | **332.492 (153)** | **0.484** | **0.055 [0.047; 0.063]** | **0.041** | **0.961** | **0.000** |
| b) *Loneliness* | | | | | | |
| Unconstrained model | 178.505 (53) |  | 0.078 [0.066; 0.091] | 0.040 | 0.957 |  |
| **Stationary model** | **182.193 (60)** | **0.895** | **0.072 [0.060; 0.084]** | **0.041** | **0.958** | **0.001** |
| c) *Depressiveness* | | | | | | |
| Unconstrained model | 139.325 (53) |  | 0.065 [0.052; 0.078] | 0.026 | 0.979 |  |
| **Stationary model** | **142.309 (60)** | **0.817** | **0.059 [0.047; 0.072]** | **0.027** | **0.980** | **0.001** |
| *Note.* RI-CLPM = Random-intercept cross-lagged panel model. df = Degrees of freedom. RMSEA = Root mean square error of approximation. SRMR = Standardized root mean square residual. CFI = Comparative fit index. Bold print indicates the selected model. | | | | | | |

| Table S8. DPM stationarity tests. | | | | | | |
| --- | --- | --- | --- | --- | --- | --- |
| *Network diversity* and | χ^2^ (df) | Δχ^2^ *p* | RMSEA [90% CI] | SRMR | CFI | ΔCFI |
| a) *Life satisfaction* | | | | | | |
| Unconstrained model | 325.665 (142) |  | 0.058 [0.049; 0.066] | 0.040 | 0.960 |  |
| **Stationary model** | **329.790 (149)** | **0.544** | **0.056 [0.048; 0.064]** | **0.041** | **0.961** | **0.001** |
| b) *Loneliness* | | | | | | |
| Unconstrained model | 177.042 (49) |  | 0.082 [0.069; 0.095] | 0.040 | 0.956 |  |
| **Stationary model** | **178.867 (56)** | **0.930** | **0.075 [0.063; 0.088]** | **0.040** | **0.958** | **0.002** |
| c) *Depressiveness* | | | | | | |
| Unconstrained model | 136.992 (49) |  | 0.068 [0.055; 0.082] | 0.024 | 0.979 |  |
| **Stationary model** | **137.332 (56)** | **0.999** | **0.061 [0.048; 0.074]** | **0.025** | **0.981** | **0.002** |
| *Note.* DPM = Dynamic panel model. df = Degrees of freedom. RMSEA = Root mean square error of approximation. SRMR = Standardized root mean square residual. CFI = Comparative fit index. Bold print indicates the selected model. | | | | | | |

| Table S9. Unstandardized RI-CLPM parameter estimates (*N* = 389). | | | | | | | | | | | |
| --- | --- | --- | --- | --- | --- | --- | --- | --- | --- | --- | --- |
| *Network diversity* and | 1. *Life satisfaction* | | |  | 1. *Loneliness* | | |  | 1. *Depressiveness* | | |
|  | Estimate | 95% CI | *p* |  | Estimate | 95% CI | *p* |  | Estimate | 95% CI | *p* |
| *Autoregressive effects* |  |  |  |  |  |  |  |  |  |  |  |
| a_1_ ND*_t_* → ND*_t+1_* | 0.475 | [-0.360; 1.309] | 0.265 |  | 0.465 | [0.093; 0.837] | 0.014 |  | 0.517 | [-0.611; 1.644] | 0.369 |
| a_2_ WB*_t_* → WB*_t+1_* | 0.524 | [0.058; 0.990] | 0.027 |  | 0.108 | [-0.275; 0.490] | 0.581 |  | 0.357 | [-0.071; 0.784] | 0.102 |
| *Cross-lagged effects* |  |  |  |  |  |  |  |  |  |  |  |
| c_1_ ND*_t_* → WB*_t+1_* | -0.247 | [-1.062; 0.568] | 0.552 |  | 0.021 | [-0.190; 0.232] | 0.844 |  | 0.042 | [-0.339; 0.423] | 0.828 |
| c_2_ WB*_t_* → ND*_t+1_* | -0.040 | [-0.345; 0.265] | 0.797 |  | 0.186 | [-0.129; 0.501] | 0.246 |  | 0.022 | [-0.561; 0.606] | 0.940 |
| *Variances* |  |  |  |  |  |  |  |  |  |  |  |
| RI*_ND_* | 0.449 | [0.180; 0.717] | 0.001 |  | 0.459 | [0.344; 0.574] | < 0.001 |  | 0.440 | [0.105; 0.774] | 0.010 |
| RI*_WB_* | 1.207 | [0.680; 1.735] | < 0.001 |  | 0.220 | [0.177; 0.263] | < 0.001 |  | 0.318 | [0.241; 0.394] | < 0.001 |
| *Correlations* |  |  |  |  |  |  |  |  |  |  |  |
| IC ND*_t1_* ↔ WB*_t1_* | -0.025 | [-0.254; 0.204] | 0.831 |  | -0.025 | [-0.052; 0.002] | 0.072 |  | -0.020 | [-0.074; 0.033] | 0.457 |
| CC ND*_t_* ↔ WB*_t_* | 0.015 | [-0.050; 0.081] | 0.645 |  | 0.000 | [-0.015; 0.015] | 0.991 |  | -0.005 | [-0.04; 0.030] | 0.776 |
| *Note.* RI-CLPM = Random-intercept cross-lagged panel model. ND = Network diversity. WB = Well-being. RI = Random intercept. IC = Initial correlation. CC = Correlated change. CFI = Comparative fit index. RMSEA = Root mean square error of approximation. SRMR = Standardized root mean square residual. | | | | | | | | | | | |

| Table S10. Unstandardized DPM parameter estimates (*N* = 389). | | | | | | | | | | | |
| --- | --- | --- | --- | --- | --- | --- | --- | --- | --- | --- | --- |
| *Network diversity* and | 1. *Life satisfaction* | | |  | 1. *Loneliness* | | |  | 1. *Depressiveness* | | |
|  | Estimate | 95% CI | *p* |  | Estimate | 95% CI | *p* |  | Estimate | 95% CI | *p* |
| *Autoregressive effects* |  |  |  |  |  |  |  |  |  |  |  |
| a_1_ ND*_t_* → ND*_t+1_* | 0.404 | [0.052; 0.757] | 0.025 |  | 0.468 | [0.115; 0.822] | 0.009 |  | 0.405 | [0.059; 0.752] | 0.022 |
| a_2_ WB*_t_* → WB*_t+1_* | 0.351 | [-0.186; 0.888] | 0.200 |  | 0.137 | [-0.272; 0.547] | 0.512 |  | 0.328 | [-0.172; 0.828] | 0.198 |
| *Cross-lagged effects* |  |  |  |  |  |  |  |  |  |  |  |
| c_1_ ND*_t_* → WB*_t+1_* | 0.017 | [-0.404; 0.438] | 0.937 |  | 0.014 | [-0.183; 0.211] | 0.887 |  | -0.069 | [-0.272; 0.133] | 0.502 |
| c_2_ WB*_t_* → ND*_t+1_* | 0.004 | [-0.143; 0.151] | 0.958 |  | 0.219 | [-0.057; 0.495] | 0.119 |  | -0.036 | [-0.287; 0.216] | 0.781 |
| *Variances* |  |  |  |  |  |  |  |  |  |  |  |
| UH*_ND_* | 0.175 | [-0.056; 0.406] | 0.138 |  | 0.159 | [-0.044; 0.362] | 0.125 |  | 0.174 | [-0.056; 0.403] | 0.139 |
| UH*_WB_* | 0.595 | [-0.507; 1.697] | 0.290 |  | 0.168 | [0.000; 0.336] | 0.050 |  | 0.149 | [-0.108; 0.405] | 0.256 |
| *Correlations* |  |  |  |  |  |  |  |  |  |  |  |
| IC ND*_t1_* ↔ WB*_t1_* | 0.058 | [-0.036; 0.153] | 0.228 |  | -0.056 | [-0.097; -0.015] | 0.007 |  | -0.040 | [-0.093; 0.012] | 0.133 |
| CC ND*_t_* ↔ WB*_t_* | 0.035 | [-0.003; 0.073] | 0.068 |  | 0.003 | [-0.011; 0.017] | 0.661 |  | -0.013 | [-0.032; 0.006] | 0.192 |
| *Note.* DPM = Dynamic panel model. ND = Network diversity. WB = Well-being. UH = Unobserved heterogeneity. IC = Initial correlation. CC = Correlated change. CFI = Comparative fit index. RMSEA = Root mean square error of approximation. SRMR = Standardized root mean square residual. | | | | | | | | | | | |

| Table S11. Changes in CLPM parameters over the duration of living alone (LSEM). | | | | | | | | |
| --- | --- | --- | --- | --- | --- | --- | --- | --- |
| *Network diversity* and | 1. *Life satisfaction* | |  | 1. *Loneliness* | |  | 1. *Depressiveness* | |
|  | *b* | *p* |  | *b* | *p* |  | *b* | *p* |
| *Autoregressive effects* |  |  |  |  |  |  |  |  |
| a_1_ ND*_t_* → ND*_t+1_* | -0.006 | 0.066 |  | -0.006 | 0.090 |  | -0.006 | 0.048 |
| a_2_ WB*_t_* → WB*_t+1_* | 0.006 | 0.110 |  | 0.002 | 0.698 |  | 0.001 | 0.690 |
| *Cross-lagged effects* |  |  |  |  |  |  |  |  |
| c_1_ ND*_t_* → WB*_t+1_* | 0.004 | 0.374 |  | 0.001 | 0.520 |  | -0.002 | 0.334 |
| c_2_ WB*_t_* → ND*_t+1_* | -0.001 | 0.414 |  | 0.000 | 0.954 |  | 0.004 | 0.174 |
| *Correlations* |  |  |  |  |  |  |  |  |
| IC ND*_t1_* ↔ WB*_t1_* | 0.005 | 0.318 |  | -0.003 | 0.144 |  | -0.005 | 0.158 |
| CC ND*_t_* ↔ WB*_t_* | 0.000 | 0.742 |  | 0.001 | 0.272 |  | 0.001 | 0.138 |
| *Note.* CLPM = Cross-lagged panel model. LSEM = Local structural equation modeling. *b* = Linear slope of model parameters across levels of the moderator. ND = Network diversity. WB = Well-being. IC = Initial correlation. CC = Correlated change. *N* = 387 because two participants had missing values in the duration of living alone. | | | | | | | | |

| Table S12. Changes in RI-CLPM parameters over the duration of living alone (LSEM). | | | | | | | | |
| --- | --- | --- | --- | --- | --- | --- | --- | --- |
| *Network diversity* and | 1. *Life satisfaction* | |  | 1. *Loneliness* | |  | 1. *Depressiveness* | |
|  | *b* | *p* |  | *b* | *p* |  | *b* | *p* |
| *Autoregressive effects* |  |  |  |  |  |  |  |  |
| a_1_ ND*_t_* → ND*_t+1_* | 0.012 | 0.578 |  | 0.013 | 0.516 |  | 0.019 | 0.484 |
| a_2_ WB*_t_* → WB*_t+1_* | 0.040 | 0.142 |  | 0.018 | 0.402 |  | -0.019 | 0.396 |
| *Cross-lagged effects* |  |  |  |  |  |  |  |  |
| c_1_ ND*_t_* → WB*_t+1_* | 0.002 | 0.998 |  | 0.007 | 0.556 |  | 0.010 | 0.416 |
| c_2_ WB*_t_* → ND*_t+1_* | -0.011 | 0.280 |  | 0.021 | 0.248 |  | 0.041 | 0.008 |
| *Correlations* |  |  |  |  |  |  |  |  |
| IC ND*_t1_* ↔ WB*_t1_* | -0.279 | 0.072 |  | 0.000 | 0.872 |  | -0.002 | 0.664 |
| CC ND*_t_* ↔ WB*_t_* | -0.001 | 0.734 |  | 0.001 | 0.236 |  | 0.003 | 0.004 |
| *Note.* RI-CLPM = Random-intercept cross-lagged panel model. LSEM = Local structural equation modeling. *b* = Linear slope of model parameters across levels of the moderator. ND = Network diversity. WB = Well-being. IC = Initial correlation. CC = Correlated change. *N* = 387 because two participants had missing values in the duration of living alone. | | | | | | | | |

| Table S13. Changes in DPM parameters over the duration of living alone (LSEM). | | | | | | | | |
| --- | --- | --- | --- | --- | --- | --- | --- | --- |
| *Network diversity* and | 1. *Life satisfaction* | |  | 1. *Loneliness* | |  | 1. *Depressiveness* | |
|  | *b* | *p* |  | *b* | *p* |  | *b* | *p* |
| *Autoregressive effects* |  |  |  |  |  |  |  |  |
| a_1_ ND*_t_* → ND*_t+1_* | 0.013 | 0.774 |  | -0.010 | 0.660 |  | 0.006 | 0.906 |
| a_2_ WB*_t_* → WB*_t+1_* | 0.026 | 0.500 |  | 0.011 | 0.702 |  | -0.009 | 0.740 |
| *Cross-lagged effects* |  |  |  |  |  |  |  |  |
| c_1_ ND*_t_* → WB*_t+1_* | 0.010 | 0.616 |  | -0.009 | 0.434 |  | 0.028 | 0.070 |
| c_2_ WB*_t_* → ND*_t+1_* | -0.009 | 0.452 |  | 0.001 | 0.972 |  | 0.057 | 0.004 |
| *Correlations* |  |  |  |  |  |  |  |  |
| IC ND*_t1_* ↔ WB*_t1_* | 0.005 | 0.366 |  | -0.004 | 0.146 |  | -0.005 | 0.098 |
| CC ND*_t_* ↔ WB*_t_* | 0.000 | 0.778 |  | -0.001 | 0.562 |  | 0.005 | 0.010 |
| *Note.* DPM = Dynamic panel model. LSEM = Local structural equation modeling. *b* = Linear slope of model parameters across levels of the moderator. ND = Network diversity. WB = Well-being. IC = Initial correlation. CC = Correlated change. *N* = 387 because two participants had missing values in the duration of living alone. | | | | | | | | |


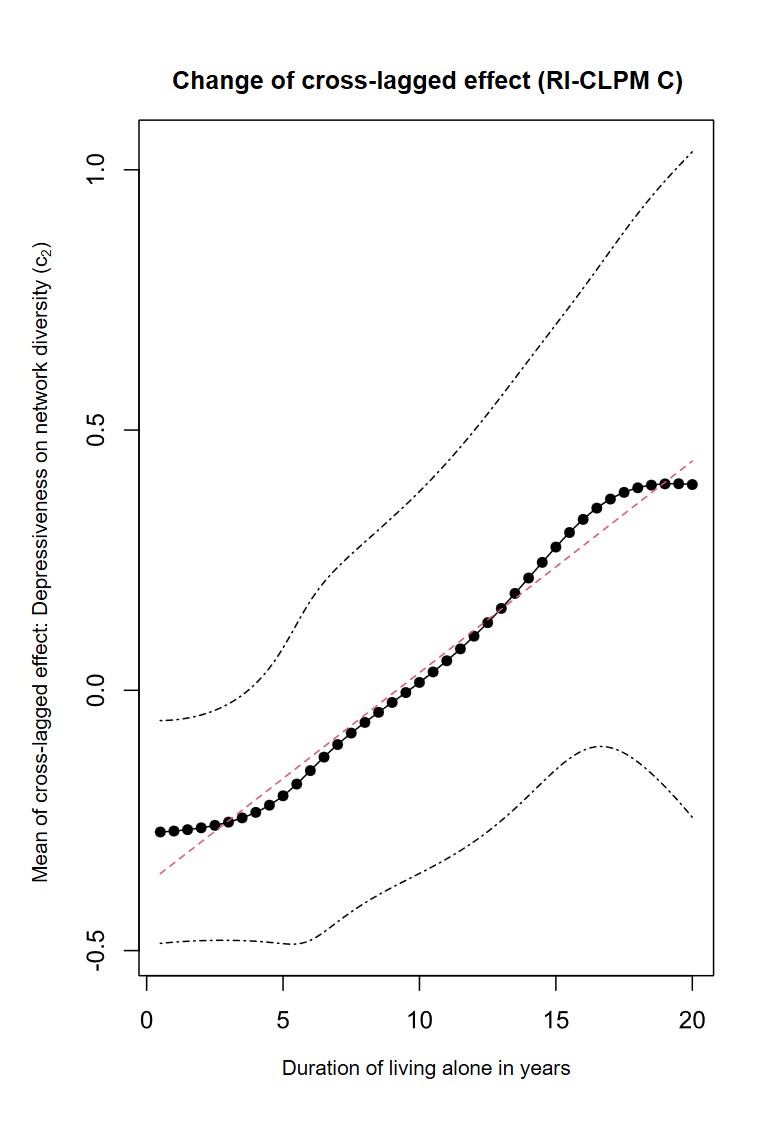


*Figure S1.* LSEM point estimates of the cross-lagged effect of depressiveness on network diversity over the duration of living alone (RI-CLPM). LSEM = Local structural equation modeling. RI-CLPM = Random-intercept cross-lagged panel model. c_2_ = Cross-lagged path. The dashed red line represents estimates based on the regression model. The dotted black line indicates LSEM point estimates. The dashed black lines display 95% confidence intervals of the LSEM estimates.


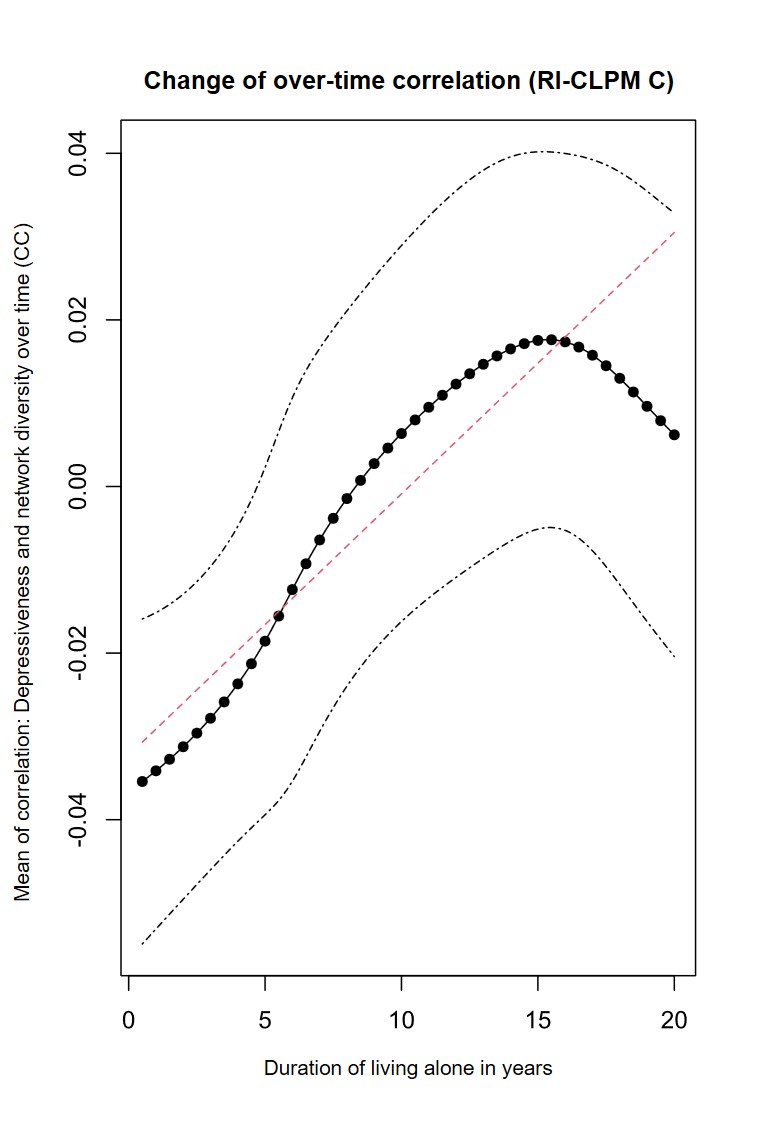


*Figure S2.* LSEM point estimates of the correlated change in network diversity and depressiveness over the duration of living alone (RI-CLPM). LSEM = Local structural equation modeling. RI-CLPM = Random-intercept cross-lagged panel model. CC = Correlated change. The dashed red line represents estimates based on the regression model. The dotted black line indicates LSEM point estimates. The dashed black lines display 95% confidence intervals of the LSEM estimates.


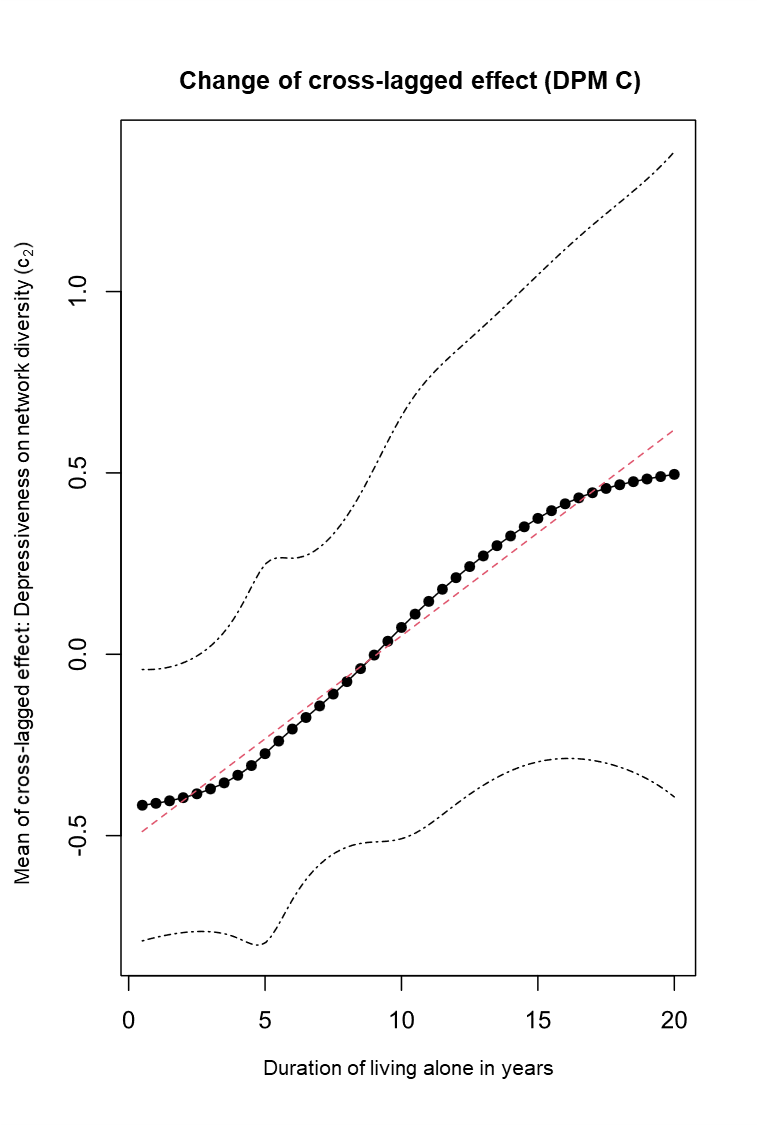


*Figure S3.* LSEM point estimates of the cross-lagged effect of depressiveness on network diversity over the duration of living alone (DPM). LSEM = Local structural equation modeling. DPM = Dynamic panel model. c_2_ = Cross-lagged path. The dashed red line represents estimates based on the regression model. The dotted black line indicates LSEM point estimates. The dashed black lines display 95% confidence intervals of the LSEM estimates.


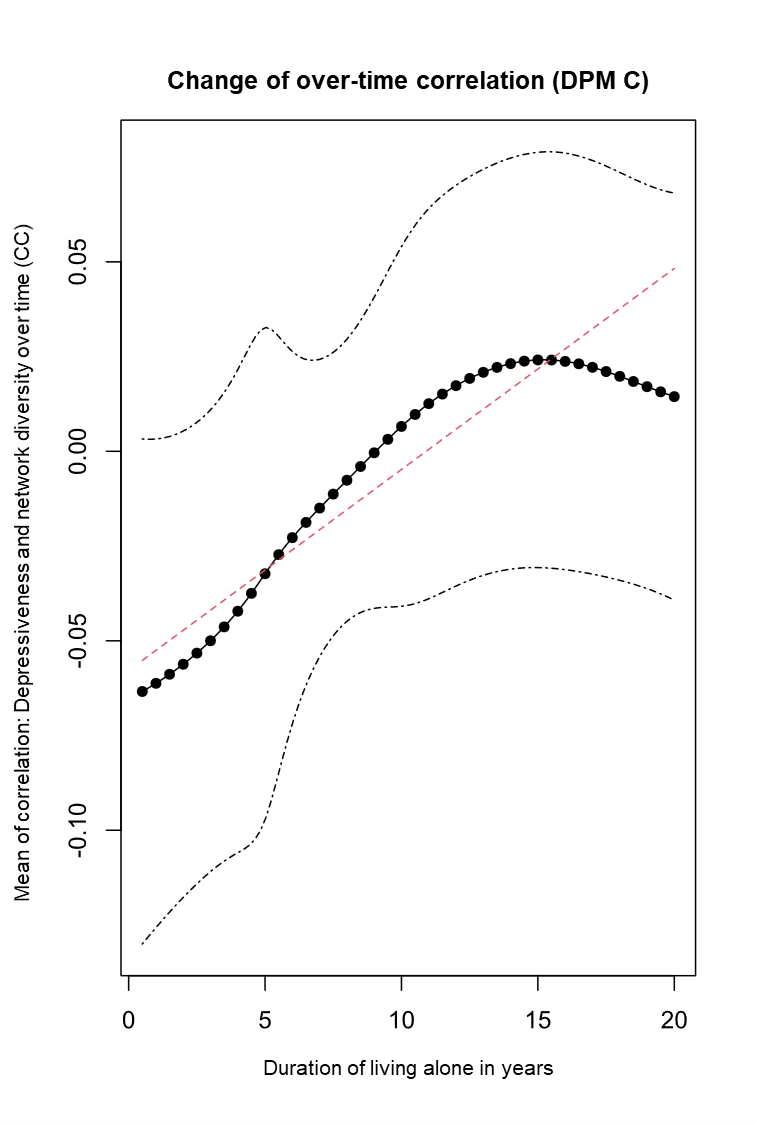


*Figure S4.* LSEM point estimates of the correlated change in network diversity and depressiveness over the duration of living alone (DPM). LSEM = Local structural equation modeling. DPM = Dynamic panel model. CC = Correlated change. The dashed red line represents estimates based on the regression model. The dotted black line indicates LSEM point estimates. The dashed black lines display 95% confidence intervals of the LSEM estimates.
